# An Ultrasensitive Lateral Flow Immunoassay Based on Metal-Organic Framework-Decorated Polydopamine for Multiple Sulfonylureas Adulteration in Functional Foods

**DOI:** 10.3390/foods12030539

**Published:** 2023-01-25

**Authors:** Zixian He, Zhiwei Liu, Haihuan Xie, Pengjie Luo, Xiangmei Li

**Affiliations:** 1 Guangdong Provincial Key Laboratory of Food Quality and Safety, College of Food Science, South China Agricultural University, Guangzhou 510642, China; 2 Wuzhou Institute for Food and Drug Control, Wuzhou 543099, China; 3 NHC Key Laboratory of Food Safety Risk Assessment, Chinese Academy of Medical Science Research Unit (No. 2019RU014), China National Center for Food Safety Risk Assessment, Beijing 100022, China

**Keywords:** sulfonylureas, metal-organic frameworks, polydopamine, lateral flow immunoassay, functional foods

## Abstract

Herein, an ultrasensitive lateral flow immunoassay (LFIA), based on metal-organic framework-decorated polydopamine (PCN-224@PDA) was first established to detect multiple sulfonylureas (SUs) in functional foods. The PCN-224@PDA was synthesized using the one-pot hydrothermal method and covalently coupled with SUs antibodies, and the coupling rate was up to 91.8%. The detection limits of the developed PCN-224@PDA-LFIA for multiple SUs in functional teas and capsules were 0.22–3.72 μg/kg and 0.40–3.71 μg/kg, and quantification limits were 0.75–8.19 μg/kg and 1.03–9.08 μg/kg, respectively. The analytical sensitivity was 128-fold higher than that of similar methods reported so far. The recovery rates ranged from 83.8 to 119.0%, with coefficients of variation of 7.6–14.4%. The parallel analysis of 20 real samples by LC-MS/MS confirmed the reliability of the proposed method. Therefore, our work offers novel, ultrasensitive, and rapid technical support for on-site monitoring of SUs in functional foods.

## 1. Introduction

Sulfonylureas (SUs) are a class of antihyperglycemic drugs, which has been widely employed to treat type 2 diabetes mellitus [1]. SUs mainly include glipizide (GP), glimepiride (GM), glyburide (GB), tolbutamide (TB), etc. Recently, it has been reported that different types of SUs are illegally adulterated in functional foods to achieve additional hypoglycemic effects. However, long-term exposure to SUs may produce cumulative toxicity, such as hypoglycemic reactions, digestive disorders, allergic reactions, etc., which poses a health threat to consumers [2,3]. Therefore, it is important to develop sensitive, rapid, and effective methods for the detection of multiple SUs in functional foods.

Currently, instrument methods have mainly been applied for the detection of SUs, such as high-performance liquid chromatography (HPLC) [4], and liquid chromatography-tandem mass spectrometry (LC-MS/MS) [5]. However, these methods require complicated operations, expensive instruments, and long analysis times, which impose restrictions on rapid screening and on-site analysis. The immunoassay methods are very conducive to overcoming the above obstacles due to their rapidity, simplicity, portability, and low cost [6]. Recently, two immunoassays, involving the enzyme-linked immunosorbent assay (ELISA) and colloidal gold-based lateral flow immunoassay (CG-LFIA), were developed for SUs detection for the first time by our group [7,8]. Compared with the ELISA, the CG-LFIA is simpler, more rapid, and has lower costs, making it particularly efficient for field detection; however, it still suffers from poor sensitivity and is generally limited to yielding qualitative or semi-quantitative results [7]. Further, the traditional CG nanoparticle shows poor brightness because of its low extinction molar coefficient and narrow particle size range, which can readily cause false-positive results when detecting complex functional food matrices [9]. Moreover, CG-antibodies (Abs) probes were fabricated by adsorbing Abs on the surface of CG via electrostatic adsorption, whereas these probes may reversibly dissociate under specific conditions, which is detrimental to the stability and reproducibility of LFIA [10,11]. Therefore, exploring a sensitive, intensely colored, and stable signal carrier to label Abs is one of the vital factors in the development of LFIA.

With advances in nanotechnology, diverse metal-organic frameworks (MOFs) have been designed and employed as favorable nanocarriers for biomolecule immobilization (i.e., antibodies (Abs), enzymes, nucleic acids) because of their abundant active functional groups, ultrahigh surface area, tunable pore size and shape, and high stability [12,13,14]. It has been reported that MOFs also could protect Abs against environmental perturbation and enhance the sensitivity of the immunoassay. For instance, Li et al. synthesized a Zr-MOF carrier to bind Abs, on this basis, Zr-MOF-LFIA was constructed, which had an 8-fold improved sensitivity compared to traditional CG-LFIA [15]. Zhand et al. introduced MOF on an ELISA plate for Abs immobilization. They found that Abs@MOF could maintain its biological activity when it was exposed to pH 5–10 and a temperature higher than 55 °C, and the fabricated MOF-based ELISA showed 225-fold more sensitivity than that of the commercial ELISA kit [16]. Accordingly, MOFs are ideal carriers for developing sensitive immunoassays. Unfortunately, there remain great challenges in constructing MOF-based colorimetric LFIA. One reason is that most MOFs are light in color, which renders the signal band of the test strip hardly visible to the naked eye. Another is that, although MOF and Abs are generally firmly bound together by a covalent coupling reaction, this procedure involves the use of EDC/NHS or glutaraldehyde for chemical crosslinking and the excessive consumption of time, which may cause reduced bioactivity or denaturation of the Abs [17].

Recently, we employed polydopamine (PDA) to modify the surface of CG to solve the problems of CG’s poor brightness and its instability when binding with Abs [18]. Our study demonstrated that the PDA coating endowed nanomaterial with better signal intensity, colloidal stability, and dispersion. Moreover, the PDA possesses abundant quinone groups that can be directly incorporated with Abs via Schiff base/Michael addition reactions, and the conjugation protocol is more rapid, efficient, and reliable than that using chemical coupling methods, thereby preserving the biological activity of immobilized Abs [19]. Inspired by these considerations, in a previous study, we successfully polymerized ZIF-8 with PDA and applied it to the detection of hydrochlorothiazide [20]. However, different MOFs have different physical and chemical properties, such as morphology, particle size, color, and biocompatibility, due to different ligands, metal ions, the ratio of ligands to metal ions, and synthesis conditions; therefore, the application range in immunoassay technology is also different [15,21,22].

In this study, a porphyrinic zirconium framework, named PCN-224, was used as the model substance, a novel PCN-224-modified PDA nanocomposite (PCN-224@PDA) was synthesized, and an ultrasensitive LFIA based on PCN-224@PDA was firstly established to detect multiple SUs in functional foods. Compared with traditional CG, PCN-224@PDA has stronger molar absorptivity, higher antibody coupling efficiency, and better environmental resistance. Therefore, our work enriches the nanocarriers in LFIA and also provides a novel, sensitive, and reliable detection method for the rapid screening of SUs in functional foods. 

## 2. Materials and Methods

### 2.1. Materials and Equipment

Glipizide (GP, 98%), glimepiride (GM, 99%), glyburide (GB, 99%), tolbutamide (TB, 99%), gliquidone (GQ, 98%), gliclazide (GL, 99%), carbutamide (CB, 98%), tolazamide (TLZ, 96%), acetohexamide (AH, 98%), chlorpropamide (CPM, 99%), glibornuride (GBN, 98%), repaglinide (RGLN, 99%), rosiglitazone (RGLT, 98%), phenformin (PF, 95%), metformin (MFM, 97%), zirconyl chloride octahydrate (ZrOCl_2_·8(H_2_O), 98%), meso-tetra (4-carboxyphenyl) porphine (H_2_TCPP, 97%), N, N-Dimethylformamide (DMF), and dopamine hydrochloride (DA, 98%) were obtained from Shanghai Aladdin Biochemical Technology Co., Ltd (Shanghai, China). The anti-SUs monoclonal antibody (anti-SUs mAb, 5 mg/mL) and coating antigen (SUs-OVA, 2 mg/mL) were prepared in our lab. 2-(N-morpholino) ethanesulfonic acid (MES, 99%) and bovine serum albumin (BSA, 98%) were purchased from Sigma-Aldrich (St. Louis, MO, USA). Nitrocellulose membranes (UniSart CN140) were received from Sartorius Stedim Biotech GmbH (Goettingen, Germany). Sample pads (SB08), polyvinylchloride backing plates (SMA31-40), and absorbent pads (CH37K) were supplied by Shanghai Liangxin Co., Ltd. (Shanghai, China).

The FIC-Q1 test strip analyzer was purchased from Suzhou Helmen Precision Instruments Co., Ltd. (Suzhou, China). The XYZ-3060 Dispensing Platform was supplied by BioDot, Inc. (Irvine, CA, USA). The CTS-300 automatic slitting machine and the ZQ-2000 strip cutter were purchased from Shanghai kinbio Tech. Co., Ltd. (Shanghai, China). The high-speed refrigerated centrifuge (GL-23M) was made by Hunan Xiangyi Technology Co., Ltd. (Changsha, China).

### 2.2. Preparation of PCN-224@PDA

**Figure 1:**
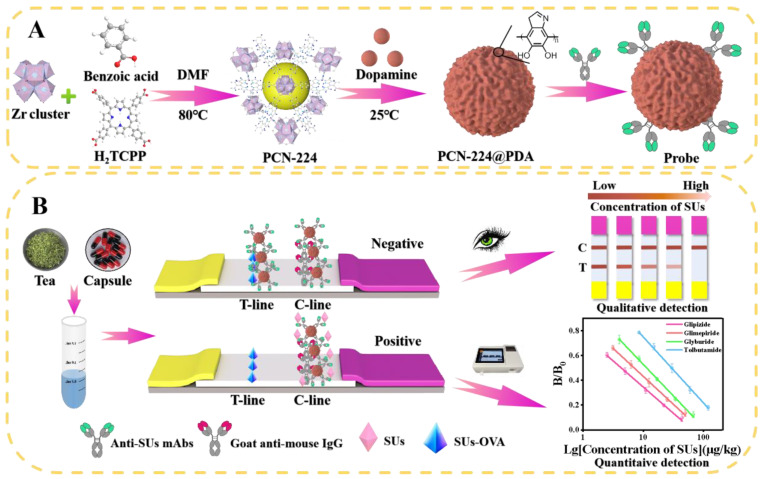
Schematic diagram of the PCN-224@PDA-LFIA for SUs detection in functional foods. (**A**) Synthesis steps for PCN-224, PCN-224@PDA, and PCN-224@PDA-Abs probes; (**B**) principle illustration of the detection strategy of PCN-224@PDA-LFIA.

The PCN-224@PDA was synthesized using the one-pot hydrothermal method (Figure 1A). First, 1.4 g of benzoic acid and 150 mg of ZrOCl_2_·8(H_2_O) were dissolved in 30 mL of DMF through sonication, and 50 mg of H_2_TCPP dissolved in 20 mL of DMF was added dropwise to the above solution. The reaction was conducted for 5 h at 80 °C with vigorous stirring. The reaction solution was washed three times with DMF, followed by washing with ethanol three times. After centrifugation at 10,000× *g* for 10 min, the precipitate (PCN-224) was collected and dried under a vacuum (80 °C, 5 h). Afterward, 24 mg of PCN-224 powder was ultrasonically dissolved in 24 mL of 75% ethanol solution. Then, 12 mg of DA and 24 mL of Tris-HCl buffer (10 mM) were added to the above solution and reacted for 12 h in a shaker (300 rpm, 25 °C). The mixture was first cleaned three times with ethanol and then washed three times with deionized water. After centrifugation at 10,000× *g* for 10 min, the dark brown product (PCN-224@PDA) was collected and dried under a vacuum (80 °C, 5 h).

### 2.3. Construction of PCN-224@PDA-Abs Probes

First, 2 mg of PCN-224@PDA was dissolved in 1 mL of MES (0.02 M, pH 6.5) through sonication; 5 μg of anti-SUs mAb was then added with stirring for 1 h in a shaker (300 rpm, 25 °C). Next, 50 μL of 10% BSA was dripped into the above mixture for 1 h of blocking. The prepared probes were collected by centrifugation (14,000× *g*, 10 min) and then resuspended in 200 μL of 0.02 M phosphate buffer (PB, pH 7.4, 0.3% PVP, 0.5% Tween^®^-20, 0.5% BSA).

### 2.4. Preparation of the PCN-224@PDA-LFIA

The LFIA platform was prepared according to our previous study [23]. For the detailed technical parameters, please refer to Table S1.

### 2.5. Sample Preparation and Detection Process

Sample preparation: Functional tea and capsule samples, which were identified as free of SUs by LC-MS/MS, were taken out of their packaging and separately ground into powder. Then, an accurately weighed amount of powder (1 ± 0.05 g) was extracted with 1 mL of methanol by vortex oscillation for 3 min. After centrifuging at 10,000× *g* for 5 min, the supernatant was filtered through a 0.22 μm organic filter membrane and then diluted five times with PB (0.02 M, pH 7.4) for subsequent analysis. 

Detection process: 120 μL of treated sample solution and 1.7 μL of PCN-224@PDA-Abs probes were mixed in the microwell, and the solution was then incubated for 3 min. The LFIA was immediately inserted into the solution. After reacting for 7 min, the LFIA was pulled out and the sample pad was removed. The qualitative results were visually determined by naked-eye observation, the quantitative results were measured by a test strip reader (Figure 1B).

### 2.6. Performance Evaluation of PCN-224@PDA-LFIA

#### 2.6.1. Sensitivity

The four most common types of SUs (i.e., GP, GM, GB, and TB), with a series of concentrations, were spiked into functional tea and capsule samples to evaluate the sensitivity of the PCN-224@PDA-LFIA. Each assay was repeated three times to determine the cut-off values, limits of detection (LODs), limits of quantitation (LOQs), and linear ranges. Based on the detection principle of PCN-224@PDA-LFIA, target analytes in the sample compete with the coating antigen to bind with limited Abs; therefore, the color signal of the T-line is inversely proportional to the target concentration (Figure 1B). The cut-off value was the threshold concentration of target analytes that could make the T-line colorless. The calibration curves were plotted using the B/B_0_ (the ratio of the T-line_absorbance_/C-line_absorbance_ value with and without SUs in the standard/sample solutions) on the *Y*-axis and the logarithm of SUs concentration on the *X*-axis. According to the calibration curves, the LODs, LOQs, and linear ranges were calculated as the concentration of target analytes, causing 10%, 20%, and 20–80% inhibition of the B/B_0_ value, respectively [24].

#### 2.6.2. Selectivity

Cross-reactions (CRs, %) were performed to evaluate the selectivity of the PCN-224@PDA-LFIA. The CR analyses of 11 types of SUs (i.e., GP, GM, GB, TB, GQ, GL, CB, TLZ, AH, CPM, and GBN) and 4 other common oral hypoglycemic drugs (i.e., RGLN, RGLT, PF, and MFM) were carried out using the developed PCN-224@PDA-LFIA and the indirect competitive enzyme-linked immunosorbent assay (icELISA), respectively. The CR rates were obtained via the following equation:

$$\mathrm{CR}\;(\%)=\frac{{\mathrm{IC}}_{50}\;(\mathrm{GP},\;\mu g/\mathrm{kg})}{{\mathrm{IC}}_{50}\;(\mathrm{Other}\;\mathrm{drugs},\;\mu g/\mathrm{kg})}\times 100\%$$

#### 2.6.3. Accuracy and Precision

The accuracy and precision of the PCN-224@PDA-LFIA were identified by recovery and the coefficient of variation (CV), respectively. Tea and capsule samples were fortified with known concentrations of GP, GM, GB, and TB, respectively. All samples were performed in triplicate on three separate days.

#### 2.6.4. Application of the PCN-224@PDA-LFIA

A total of 20 functional foods (10 teas, 10 capsules) were purchased from a local market. Each sample was tested three times using PCN-224@PDA-LFIA and LC-MS/MS, respectively. The LC-MS/MS parameters are described in the Supporting Information, and the MS/MS details can be found in Table S2. 

### 2.7. Statistical Analysis

Each test was conducted with three replicates (*n* = 3). Data were presented as mean ± standard deviation (SD). GraphPad Prism 8.0 and Origin 2021 were used for statistical analysis. The significance level was determined at *p* < 0.01.

## 3. Results and Discussion

### 3.1. Characterization of PCN-224 and PCN-224@PDA

**Figure 2:**
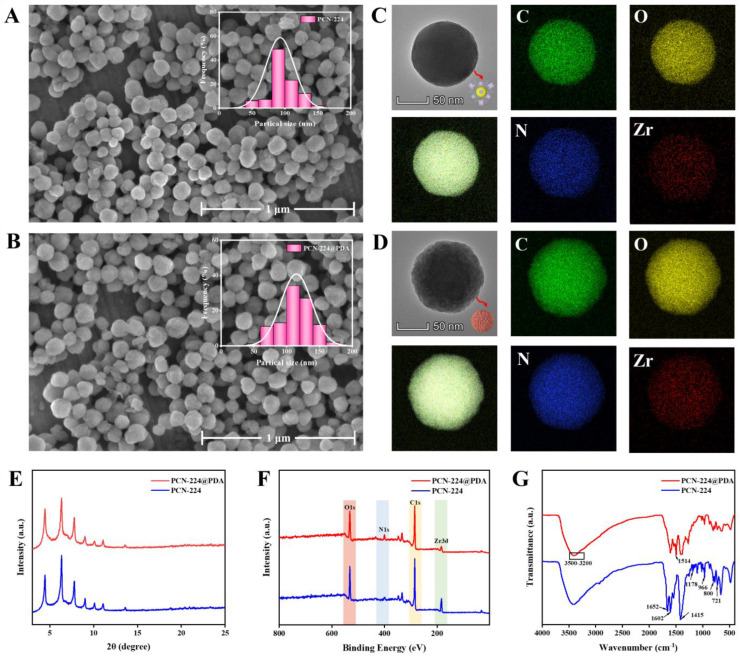
Characterization of PCN-224 and PCN-224@PDA. SEM images of (**A**) PCN-224 and (**B**) PCN-224@PDA, respectively. TEM images and EDS elemental mappings of (**C**) PCN-224 and (**D**) PCN-224@PDA, respectively; (**E**–**G**) XRD, XPS, and FT-IR spectra of PCN-224 and PCN-224@PDA, respectively.

The results of the scanning electron microscopy (SEM) (Figure 2A,B) illustrated that PCN-224 and PCN-224@PDA were homogeneous in sphericity with a mean particle size of 100 and 120 nm, respectively, indicating that the PDA layer with a thickness of 20 nm was formed on the PCN-224 surface. The transmission electron microscopy (TEM) (Figure 2C,D) showed that PCN-224 presented a smooth surface, while that of the PCN-224@PDA was rough, which further revealed that PCN-224 was successfully modified by PDA. Energy dispersive spectrometer (EDS) elemental mapping analyses displayed that PCN-224 preserved native three-dimensional structures after modification with PDA coating; the four elements of Zr, C, O, and N were still presented and evenly distributed within the PCN-224 or PCN-224@PDA. The X-ray diffraction (XRD) pattern (Figure 2E) of PCN-224@PDA matched well with the characteristic diffraction peaks of PCN-224, which suggested that the crystallinity of the PCN-224 was not altered upon the surface modification with PDA. The X-ray photoelectron spectroscopy (XPS) spectra (Figure 2F) showed that the element of Zr 3d was obviously attenuated in the spectrum of PCN-224@PDA compared with that of the PCN-224, which was due to the shielding effect of the PDA coating. The XPS high-resolution spectra in the region of each element are shown in Figure S2. Additionally, the Fourier-transform infrared (FT-IR) spectrum of PCN-224@PDA (Figure 2G) did not completely match that of the PCN-224 and displayed two new characteristic peaks of indole and N-H/O-H at 1514 cm^−1^ and 3200–3500 cm^−1^, respectively [25], which further verified that the PCN-224@PDA was successfully synthesized.

### 3.2. Synthesis Optimization of PCN-224@PDA

#### 3.2.1. Optimization of the pH Value for the Self-Polymerization of DA

**Figure 3:**
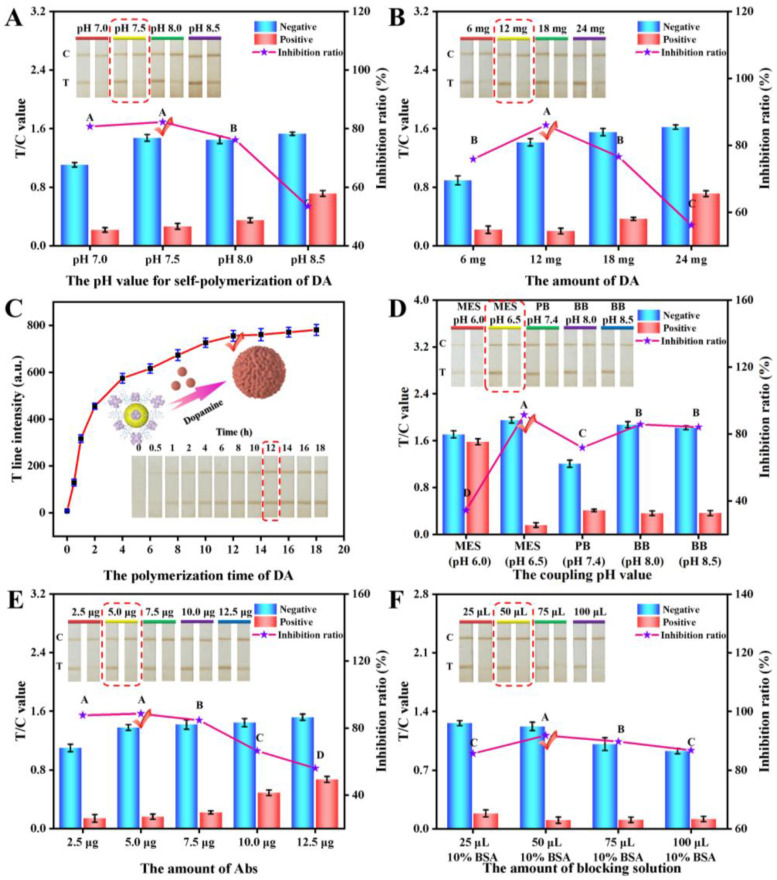
Synthesis optimization of PCN-224@PDA, including (**A**) the effect of the pH value for the self-polymerization of DA, (**B**) the effect of the DA amount, and (**C**) the effect of the polymerization time of DA. Optimization of the key technical parameters of PCN-224@PDA-ICA, including (**D**) the effect of the coupling pH value, (**E**) the effect of the Ab amount, and (**F**) the effect of the amount of blocking solution. The inhibition ratio was obtained through the formula: inhibition ratio (%) = (1 − (T⁄C) _positive_/(T⁄C) _negative_) × 100%. The concentration of GP was fixed at 5 ng/mL (in 0.02 M PB buffer) for positive results. Different letters represent statistical differences (*p* < 0.01).

The pH of the solution is a critical parameter influencing the polymerization of DA and its binding to the surface of PCN-224. In an acidic environment, DA is not easy to polymerize on the surface of MOF material, and a high pH value can easily cause DA aggregation. Therefore, in this study, we evaluated the synthesis effect in the pH range of 7–8.5 [26]. As seen in Figure 3A, the T/C signal increased gradually with the pH value, while the inhibition rate of the test strip decreased significantly when the pH exceeded 7.5. Therefore, pH 7.5 was selected as the optimal condition.

#### 3.2.2. Optimization of the Amount of DA

The amount of DA is intimately associated with the thickness of the PDA coating, dispersibility, and color intensity of nanoparticles [27]. Figure 3B displays the effect of the DA amount on the performance of the test strip, which revealed that the signal strength increased with a higher amount of DA, while the inhibition rate exhibited a significant decrease. When the amount of DA used was 12 mg, the test strip acquired a desirable color intensity and inhibition rate. Additionally, we found that further increasing the DA amount beyond 12 mg could cause the resulted PCN-224@PDA solution to form condensates, resulting in poor dispersion stability. Therefore, the optimal amount of DA was selected as 12 mg.

#### 3.2.3. Optimization of the Polymerization Time of DA

The control of the polymerization time of DA on the PCN-224 permits control of the adhesion strength of the PDA coating and the optical intensity of nanoparticles [20]. The results showed (Figure 3C) that the color intensity of the T-line increased with the prolongation of the polymerization time. When the time increased beyond 12 h, the signal intensity of the T-line did not continue to increase, suggesting that the DA polymerization reaction reached saturation on the surface of the PCN-224. Therefore, the optimal polymerization time was chosen as 12 h.

### 3.3. Optimization of Key Technical Parameters of PCN-224@PDA-LFIA

To guarantee good performance of the PCN-224@PDA-LFIA, several pivotal parameters were evaluated below, including the coupling pH value, the amount of Abs, the blocking solution, and the sample dilution ratio.

#### 3.3.1. Optimization of the Coupling pH Value

The proper coupling pH value plays an essential role in the conjugation of PCN-224@PDA to Abs, which contributes to achieving robust immobilization of Abs, fully exposing its antigen-binding fragment, and enhancing antigen recognition capability [6]. Figure 3D shows the influence of the different coupling pH values, which indicated that when the coupling pH of 6.5 was employed, the maximum color strength and inhibition rate of the test strip were acquired compared with that of other pH conditions. Therefore, Abs demonstrated the highest activity in binding to PCN-224@PDA at pH 6.5, with a coupling ratio of up to 91.8% (Figure S1).

#### 3.3.2. Optimization of the Amount of Abs

In the competition-type immunoreaction, the limited Abs amount should be used for obtaining the highest sensitivity [28,29]. The results (Figure 3E) showed that an increasing trend in the signal strength of the T-line was observed with increasing Abs amounts. However, the inhibition rate began to decline when more than 5.0 μg of Abs was applied, indicating that excessively labeled Abs was not favorable for the sensitivity of the developed PCN-224@PDA-LFIA. Therefore, the optimal amount of Abs was 5.0 μg.

#### 3.3.3. Optimization of the Amount of Blocking Solution

A blocking solution can block the nonspecific binding sites on the nanoparticles and, at the same time, improve the stability of the nanoprobe [30]. In this study, different amounts of 10% BSA were used for comparison. The results (Figure 3F) showed that the color intensity decreased with increasing BSA amounts, while too low or too high an amount of BSA was not conducive to the inhibition rate. When the amount of BSA was 50 μL, the test strip acquired desirable signal intensity and a good inhibitory effect. Therefore, the best amount of the blocking agent was 50 μL.

#### 3.3.4. Optimization of the Sample Dilution Ratio

Direct dilution of the treated sample solution is the simplest and most straightforward strategy to remove sample matrix interference [31,32]. As shown in Figure S3A,B, with an increasing sample dilution ratio, the background interference caused by tea and capsules significantly decreased, and the best color development and inhibition performance were obtained at a dilution ratio of 1:4. Therefore, the treated tea and capsule sample solution should be diluted in a ratio of 1:4.

### 3.4. Performance Evaluation of PCN-224@PDA-LFIA

#### 3.4.1. Sensitivity

**Figure 4:**
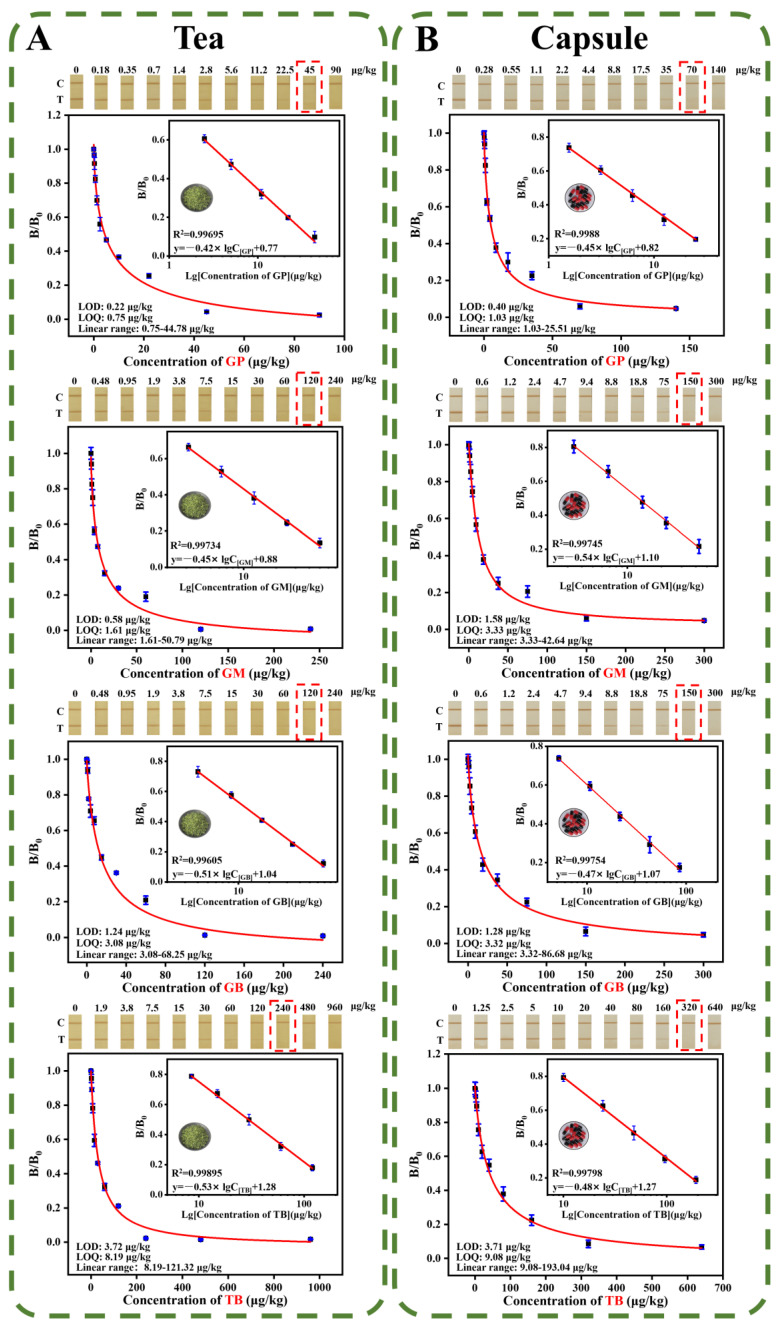
Performance evaluation of the PCN@PDA-LFIA. The cut-off value and calibration curves of PCN@PDA-LFIA for detecting GP/GM/GB/TB in (**A**) functional tea samples and (**B**) functional capsule samples, respectively.

As shown in Figure 4, the cut-off values for GP/GM/GB/TB in functional tea and capsule samples were 45/120/120/240 and 70/150/150/320 μg/kg, the LODs were 0.22/0.58/1.24/3.72 and 0.40/1.58/1.28/3.71 μg/kg, the LOQs were 0.75/1.61/3.08/8.19 and 1.03/3.33/3.32/9.08 μg/kg, the linear ranges were 0.75–44.78/1.61–50.79/3.08–68.25/8.19–121.32 and 1.03–25.51/3.33–42.64/3.32–86.68/9.08–193.04 μg/kg, respectively.

#### 3.4.2. Selectivity

The selectivity results are shown in Table S3. SUs Abs exhibited a different CR to GP (100%), GB (100%), GM (83.3%), GQ (68.2%), TB (23.1%), CB (17%), etc. This is because the SUs have a benzene ring connected to a sulfonylurea group as their basic structure, and also contain two prosthetic groups, namely a sulfo group and a urea group. The difference in the two prosthetic groups makes the exposure of the basic structure, that is, the antigen recognition site, different. Therefore, the SUs Abs prepared based on GP-BSA have different recognition abilities for different drugs. SUs Abs have no CR with other oral hypoglycemic drugs (RGLN, RGLT, PF, MFM). This is because the structure of the above drugs is completely different from SUs. The selectivity results of PCN-224@PDA-LFIA were mostly consistent with that of icELISA. The developed method has a CR of more than 2.7% for 9 kinds of SUs drugs, demonstrating that the method can detect 9 kinds of SUs drugs simultaneously.

#### 3.4.3. Accuracy and Precision

As shown in Table 1, the recoveries of GP/GM/GB/TB in functional tea and capsule samples ranged from 100.3–114.0%/85.3–91.5%/108.8–115.7%/90.0–112.8% and 106.0–119.0%/105.8–119.0%/88.0–113.7%/83.8–108.2%, respectively, with corresponding CVs of 11.4–14.4%/9.9–12.6%/7.6–11.0%/9.1–12.4% and 8.0–13.2%/9.3–11.8%/8.6–11.4%/7.6–11.1%, respectively. These results manifested that the developed PCN-224@PDA-LFIA showed good accuracy and reproducibility.

#### 3.4.4. Application of the PCN-224@PDA-LFIA

As shown in Table S4, all 20 real teas and capsules were tested as negative samples by the established PCN-224@PDA-LFIA and LC-MS/MS. It was possible that the occurrence of such adulterations was very low, recently, under the effective supervision of the regulatory authorities, or the total sample volumes collected were limited. This, however, does not imply that there would be no adulteration of SUs in functional foods; illicitly added SUs remain a serious problem and continue to threaten human health [1,2]. Thus, follow-up studies will continue to focus on screening adulterated samples. In addition, the detection results of PCN-224@PDA-LFIA were consistent with those of LC-MS/MS, with no false negative or false positive results, indicating that our developed method is reliable.

### 3.5. Comparison of the Methods for the Detection of SUs

The available methods for detecting SUs were compared in Table 2. Currently, the detection of SUs in functional foods is primarily based on instrument methods [4,5,33,34,35,36]; only two immunoassay methods (i.e., ELISA and LFIA) for the detection of SUs have been reported [7,8]. The sensitivity of our developed PCN-224@PDA-LFIA was comparable to that of the conventional ELISA method and showed 128-fold more sensitivity than that of the previously reported CG-LFIA. Additionally, compared to the instrument analysis, the pretreatment of tea and capsule samples only needed simple and rapid extraction, dilution, and filtration, some complex operations, such as ultrasound and nitrogen blowing concentration, could be avoided. The whole process takes only 18 min, with an average time savings of half an hour compared to instrument methods. Moreover, the cost of the LFIA assay was only $0.032 per test, which was about 1/20 the price of the instrument methods. Overall, this work was clearly better than the reported immunoassay and instrument methods due to its simplicity, rapidity, sensitivity, and cost-effectiveness.

## 4. Conclusions

In this study, an ultrasensitive LFIA based on PCN-224@PDA was first established for the detection of multiple SUs in functional foods. The PCN-224@PDA nanocomposites not only possessed excellent biocompatibility and high coupling rates with Abs but also exhibited good anti-interference capability in a complex matrix. The developed PCN-224@PDA-LFIA could simultaneously recognize 9 kinds of SUs in functional teas and capsules with LODs of 0.22–3.72 μg/kg and 0.40–3.71 μg/kg, respectively. Furthermore, the analytical sensitivity was 128-fold better than that of similar methods reported so far. The developed method displayed high sensitivity, good accuracy, precision, and satisfactory reliability, which can satisfy the requirement of regulatory agencies for SUs adulteration detection. Therefore, our work offers novel, ultrasensitive, and rapid technical support for the on-site monitoring of SUs in functional foods.

## Figures and Tables

**Table 1:** Recovery of SUs in functional tea and capsule by PCN-224@PDA-LFIA (n = 3).

Analytes	Tea				Capsule			
	Spiked Level (μg/kg)	Detected Level (μg/kg)	Recovery (%)	CV (%)	Spiked Level (μg/kg)	Detected Level (μg/kg)	Recovery (%)	CV (%)
GP	1	1.14 ± 0.13	114.0	11.4	1	1.06 ± 0.14	106.0	13.2
	2	2.02 ± 0.29	101.0	14.4	2	2.38 ± 0.19	119.0	8.0
	8	8.02 ± 1.11	100.3	13.8	10	11.63 ± 1.22	116.3	10.5
GM	2	1.83 ± 0.23	91.5	12.6	4	4.41 ± 0.43	110.3	9.8
	4	3.54 ± 0.42	88.5	11.9	8	8.46 ± 0.79	105.8	9.3
	20	17.06 ± 1.69	85.3	9.9	35	41.63 ± 4.91	119.0	11.8
GB	4	4.35 ± 0.48	108.8	11.0	4	3.52 ± 0.40	88.0	11.4
	8	9.06 ± 0.77	113.3	8.5	8	7.53 ± 0.80	94.1	10.6
	30	34.72 ± 2.64	115.7	7.6	35	39.79 ± 3.42	113.7	8.6
TB	8	8.88 ± 0.85	111.0	9.6	10	9.69 ± 0.78	96.9	8.0
	16	18.05 ± 2.24	112.8	12.4	20	16.76 ± 1.28	83.8	7.6
	80	71.99 ± 6.56	90.0	9.1	100	108.21 ± 12.01	108.2	11.1

**Table 2:** Performance comparison of the developed PCN-224@PDA-LFIA with other methods for SUs detection.

Methods	Sample	Sample Pretreatment	Detection Time	Detection Limit (μg/kg)	Quantification Limit (μg/kg)	Cut-Off Value (μg/kg)	References
HPLC ^a^	Herbal, Health foods	Ultrasound-assisted extraction, centrifugation, filtration (0.22 μm filter), dilution	43 min	216	432	-	[4]
HPLC	Milk powder and capsules	Ultrasound-assisted biphasic extraction, Nitrogen blowing concentration, redissolution	65 min	2450	8170	-	[33]
UPLC ^b^	Tablets, capsules, and particles	Ultrasound-assisted extraction, dilution, filtration (0.45 μm filter)	25 min	2.7	-	-	[34]
Ion-Pair LC ^c^	Tablets, pills, granules, and capsules	Ultrasound-assisted extraction, dilution	47 min	330	1010	-	[35]
LC-MS/MS ^d^	tablets, pills, and capsules	Ultrasound-assisted extraction, dilution, filtration (0.22 μm filter)	35 min	0.5	15	-	[5]
LC-Q/TOF ^e^	tablets, pills, and capsules	Shake extraction, centrifugation, purification, centrifugation, Nitrogen blowing concentration, redissolution	47 min	1.05	-	-	[36]
ELISA ^f^	pills and capsules	Extraction, centrifugation, filtration (0.22 μm filter), dilution	90 min	0.3	0.7	-	[8]
CG-LFIA ^g^	teas	Extraction, centrifugation, filtration (0.22 μm filter), dilution	18 min	32	-	96	[7]
PCN-224@ PDA-LFIA	Functional teas and capsules	Extraction, centrifugation, filtration (0.22 μm filter), dilution	10 min	Tea: 0.22(GP); Capsule: 0.4 (GP)	Tea: 0.75(GP); Capsule: 1.03(GP)	Tea: 45(GP); Capsule: 70 (GP)	This work

HPLC ^a^, high-performance liquid chromatography; UPLC ^b^, ultra-high performance liquid chromatography; Ion-Pair LC ^c^, ion-pair liquid chromatography; LC-MS/MS ^d^, liquid chromatography-tandem mass spectrometry; LC-Q/TOF ^e^, liquid chromatography/time-of-flight mass spectrometry; ELISA ^f^, enzyme-linked immunosorbent assay; CG-LFIA ^g^, colloidal gold-based lateral flow immunoassay.

## Data Availability

Data is contained within the article or supplementary material.

## Supplementary Materials

The following supporting information can be downloaded at: https://www.mdpi.com/article/10.3390/foods12030539/s1, Figure S1: The calibration curve of anti-SUs mAbs; Figure S2: Corresponding XPS high-resolution spectra in the regions of C 1s, N 1s, and O 1s. A-C: PCN-224; D-F: PCN-224@PDA. Compared with the PCN-224, the PCN-224@PDA displayed higher or lower binding energy in the regions of C 1s, N 1s, and O 1s. Further, the XPS spectrum in the region of C 1s for PCN-224@PDA showed a new π–π band at 289.0 eV, which was attributed to the aromatic ring of PDA loaded onto the PCN-224 surface through π–π stacking; Figure S3: The influence of sample dilution factor on test strip results. A: Tea sample; B: Capsule sample. The concentration of GP was fixed at 45 μg/kg and 70 μg/kg in tea and capsule samples, respectively. Different letters represent statistical differences (*p* < 0.01); Table S1: Working conditions of the PCN-224@PDA-LFIA; Table S2: The optimal MS/MS parameters for the detection of SUs; Table S3: Cross-reactivity of icELISA and PCN-224@PDA-LFIA (*n* = 3); Table S4: Determination of SUs in commercial functional tea and capsule samples by PCN-224@PDA-LFIA and LC-MS/MS (*n* = 3).

## References

[1] Li Y., Hu Y., Ley S., Rajpathak S., Hu F. (2014). Sulfonylurea use and incident cardiovascular disease among patients with type 2 diabetes: Prospective cohort study among women. Diabetes Care.

[2] Garcin L., Mericq V., Fauret-Amsellem A., Cave H., Polak M., Beltrand J. (2020). Neonatal diabetes due to potassium channel mutation: Response to sulfonylurea according to the genotype. Pediatr. Diabetes.

[3] Cao J., Jiang Q., Li R., Xu Q., Li H. (2019). Nanofibers mat as sampling module of direct analysis in real time mass spectrometry for sensitive and high-throughput screening of illegally adulterated sulfonylureas in antidiabetic health-care teas. Talanta.

[4] Jin P., Xu S., Xu W., He X., Kuang Y., Hu X. (2020). Screening and quantification of fourteen synthetic antidiabetic adulterants in herbal pharmaceuticals and health foods by HPLC and confirmation by LC-Q-TOF-MS/MS. Food Addit. Contam. A.

[5] Kim N., Yoo G., Kim K., Lee J., Park S., Baek S., Kang H. (2019). Development and validation of an LC-MS/MS method for the simultaneous analysis of 26 anti-diabetic drugs in adulterated dietary supplements and its application to a forensic sample. Anal. Sci. Technol..

[6] Liu Z., Hua Q., Wang J., Liang Z., Li J., Wu J., Shen X., Lei H., Li X. (2020). A smartphone-based dual detection mode device integrated with two lateral flow immunoassays for multiplex mycotoxins in cereals. Biosens. Bioelectron..

[7] Xie H., Li Y., Wang J., Lei Y., Koidis A., Li X., Shen X., Xu Z., Lei H. (2022). Broad-specific immunochromatography for simultaneous detection of various sulfonylureas in adulterated multi-herbal tea. Food Chem..

[8] Li Z., Xie H., Fu T., Li Y., Shen X., Li X., Lei Y., Yao X., Koidis A., Liu Y. (2022). Complementary strategy enhancing broad-specificity for multiplexed immunoassay of adulterant sulfonylureas in functional food. Biosensors.

[9] Li X., Chen X., Wu X., Wang J., Liu Z., Sun Y., Shen X., Lei H. (2019). Rapid detection of adulteration of dehydroepiandrosterone in slimming products by competitive indirect enzyme-linked immunosorbent assay and lateral flow immunochromatography. Food Agric. Immunol..

[10] Nardo F., Cavalera P., Baggiani C., Giovannoli C., Anfossi L. (2019). Direct vs mediated coupling of antibodies to gold nanoparticles: The case of salivary cortisol detection by lateral flow immunoassay. ACS Appl. Mater. Inter..

[11] Chen J., Luo P., Liu Z., He Z., Pang Y., Lei H., Xu Z., Wang H., Li X. (2020). Rainbow latex microspheres lateral flow immunoassay with smartphone-based device for simultaneous detection of three mycotoxins in cereals. Anal. Chim. Acta.

[12] Liu Z., Hua Q., Wang J., Liang Z., Zhou Z., Shen X., Lei H., Li X. (2020). Prussian blue immunochromatography with portable smartphone-based detection device for zearalenone in cereals. Food Chem..

[13] Liao F., Lo W., Hsu Y., Wu C., Wang S., Shieh F., Morabito J., Chou L., Wu K., Tsung C. (2017). Shielding against unfolding by embedding enzymes in metal–organic frameworks via a de novo approach. J. Am. Chem. Soc..

[14] Xu Z., Long L., Chen Y., Chen M., Cheng Y. (2021). A nanozyme-linked immunosorbent assay based on metal–organic frameworks (MOFs) for sensitive detection of aflatoxin B1. Food Chem..

[15] Li R., Bu T., Zhao Y., Sun X., Wang Q., Tian Y., Bai F., Wang L. (2020). Polydopamine coated zirconium metal-organic frameworks-based immunochromatographic assay for highly sensitive detection of deoxynivalenol. Anal. Chim. Acta.

[16] Zhand S., Razmjou A., Azadi S., Bazaz S., Shrestha J., Jahromi M., Warkiani M. (2020). Metal–organic framework-enhanced ELISA platform for ultrasensitive detection of PD-L1. ACS Appl. Bio Mater..

[17] Liu S., Dou L., Yao X., Zhang W., Zhao B., Wang Z., Ji Y., Sun J., Xu B., Zhang D. (2020). Polydopamine nanospheres as high-affinity signal tag towards lateral flow immunoassay for sensitive furazolidone detection. Food Chem..

[18] Pang Y., Zhao S., Liu Z., Chen J., Yang Z., He Z., Shen X., Lei H., Li X. (2022). An enhanced immunochromatography assay based on colloidal gold-decorated polydopamine for rapid and sensitive determination of gentamicin in animal-derived food. Food Chem..

[19] Chen R., Dong Y., Hong F., Zhang X., Wang X., Wang J., Chen Y. (2022). Polydopamine nanoparticle-mediated, click chemistry triggered, microparticle-counting immunosensor for the sensitive detection of ochratoxin A. J. Hazard Mater..

[20] Liu Z., He Z., Wu J., Lin H., Deng Y., Shen X., Lei H., Li X. (2023). Facile immunochromatographic assay based on metal–organic framework-decorated polydopamine for the determination of hydrochlorothiazide adulteration in functional foods. Food Chem..

[21] Tian Y., Bu T., Zhang M., Sun X., Jia P., Wang Q., Liu Y., Bai F., Zhao S., Wang L. (2021). Metal-polydopamine framework based lateral flow assay for high sensitive detection of tetracycline in food samples. Food Chem..

[22] Zhang G., Deng S., Fang B., Zhang G., Lai X., Su L., He W., Lai W. (2022). Lateral flow immunoassay based on polydopamine-coated metal-organic framework for the visual detection of enrofloxacin in milk. Anal. Bioanal. Chem..

[23] Liang J., Liu Z., Xie H., Fang Y., Quan Q., Shen X., Lei H., Xu Z., Li X. (2020). Ultrasensitive magnetic assisted lateral flow immunoassay based on chiral monoclonal antibody against R-(−)-salbutamol of broad-specificity for 38 β-agonists detection in swine urine and pork. J. Agric. Food Chem..

[24] Guan T., Shen Y., Jiang Z., Zhao Y., Liang Z., Liu Y., Shen X., Li X., Xu Z., Lei H. (2022). An ultrasensitive microfluidic chip-based immunoassay for multiplex determination of 11 PDE-5 inhibitors in adulterated health foods. Sens. Actuat. B-Chem..

[25] Ren R., Cai G., Yu Z., Zeng Y., Tang D. (2018). Metal-polydopamine framework: An innovative signal-generation tag for colorimetric immunoassay. Anal. Chem..

[26] Feng J., Fan H., Zha D., Wang L., Jin Z. (2016). Characterizations of the formation of polydopamine-coated halloysite nanotubes in various pH environments. Langmuir.

[27] Zhou J., Xiong Q., Ma J., Ren J., Messersmith P., Chen P., Duan H. (2016). Polydopamine-enabled approach toward tailored plasmonic nanogapped nanoparticles: From nanogap engineering to multifunctionality. ACS Nano..

[28] Bai F., Bu T., Li R., Zhao S., He K., Li M., Zhang H., Zhang Y., Zhang L., Wang Y. (2022). Rose petals-like Bi semimetal embedded on the zeolitic imidazolate frameworks based-immunochromatographic strip to sensitively detect acetamiprid. J. Hazard. Mater..

[29] Li X., Chen X., Liu Z., Wang J., Hua Q., Liang J., Shen X., Xu Z., Lei H., Sun Y. (2021). Latex microsphere immunochromatography for quantitative detection of dexamethasone in milk and pork. Food Chem..

[30] Liu Z., Chen J., Zhao S., Pang Y., Shen X., Lei H., Li X. (2022). Immunochromatographic assays based on three kinds of nanoparticles for the rapid and highly sensitive detection of tylosin and tilmicosin in eggs. Microchim. Acta.

[31] Hua Q., Liu Z., Wang J., Liang Z., Zhou Z., Shen X., Lei H., Li X. (2022). Magnetic immunochromatographic assay with smartphone-based readout device for the on-site detection of zearalenone in cereals. Food Control.

[32] Jiang J., Luo P., Liang J., Shen X., Lei H., Li X. (2020). A highly sensitive and quantitative time resolved fluorescent microspheres lateral flow immunoassay for streptomycin and dihydrostreptomycin in milk, honey, muscle, liver, and kidney. Anal. Chim. Acta.

[33] Liu Y., Pi J., Jin P., Xie X., Zhang Y., Yue Z., Mai X., Fan H., Zhang W. (2020). Multi-dimensional fingerprint profiling analysis for screening and quantification of illegal adulterated antidiabetics in hypoglycemic health products by aqueous two-phase extraction and multi-wavelength detection. J. Chromatogr. A.

[34] Du Y., Li Q., Wu C., Zhang Y. (2015). Rapid screening and quantitative detection of 11 illegally added antidiabetics in health care products by ultra-performance liquid chromatography-quadrupole/electrostatic field orbitrap high resolution mass spectrometry. Chin. J. Chromatogr..

[35] Cui M., Li N., Qin F., Li F., Xiong Z. (2010). Simultaneous determination of 14 illegal adulterants in Chinese proprietary medicines using reversed-phase ion-pair LC. Chromatographia.

[36] Ki N., Hur J., Kim B., Kim K., Moon B., Oh H., Hong J. (2019). Rapid screening of sulfonamides in dietary supplements based on extracted common ion chromatogram and neutral loss scan by LC-Q/TOF-mass spectrometry. J. Food Drug. Anal..

