# Supplementary material for: An Ultrasensitive Lateral Flow Immunoassay Based on Metal-Organic Framework-Decorated Polydopamine for Multiple Sulfonylureas Adulteration in Functional Foods
Source: Foods. 2023 Jan 25;12(3):539. doi: 10.3390/foods12030539 (PMC9914140; doi:10.3390/foods12030539)
Supplement: Supplementary file 1 [file foods-12-00539-s001.zip › foods-2144748-supplementary.pdf]

Supplementary Materials

An Ultrasensitive Lateral Flow Immunoassay Based on Metal-Organic Framework-Decorated Polydopamine for Multiple Sulfonylureas Adulteration in Functional Foods

Zixian He [1,†], Zhiwei Liu [1, †], Haihuan Xie [2], Pengjie Luo [3,*] and Xiangmei Li [1,*]

[1] Guangdong Provincial Key Laboratory of Food Quality and Safety, College of Food Science, South China Agricultural University, Guangzhou 510642, China
[2] Wuzhou Institute for Food and Drug Control, Wuzhou 543099, China
[3] NHC Key Laboratory of Food Safety Risk Assessment, Chinese Academy of Medical Science Research Unit (No. 2019RU014), China National Center for Food Safety Risk Assessment, Beijing 100022, China
* Correspondence: pengjieluo@cfsa.net.cn (P.L.); lixiangmei12@163.com (X.L.); Tel.: +86-20-8528-3925 (X.L.)
† These authors contributed equally to this work.

1. LC-MS/MS analysis

The confirmation test was performed on LC-MS/MS (AB QTRAP4500 triple quadrupole mass spectrometer). Agilent Poroshell HPH-C18 (2.1 × 150 mm, 4 μm) was used to resolve the analytes. The column temperature was set to 40 °C. Mobile phase A was an aqueous solution containing 0.1% formic acid, and B was a 100% acetonitrile. All mobile phases were sonicated for 5 min before use. The gradient elution was 0~7 min, 20%~90% B; 7~8 min, 90% B; 8~10 min, 10% B. The mobile phase flow rate was set at 300 μL/min and the injection volume of the sample was 10 μL. For details of MS/MS parameters, please refer to Table S2.

2. Quantification of the coupling rate of PCN-224@PDA to Abs

The coupling rate of PCN-224@PDA to Abs was calculated by the following formula: Coupling rate (%) $= \left(1 - \frac{\mathrm{Mr}}{\mathrm{Ma}}\right) \times 100\%$. M_a was the amount of Abs added for coupling reaction. M_r was the remaining amount of Abs in supernatant after the preparation of PCN-224@PDA-Abs probes; M_r was calculated by multiplying the Abs concentration and total volume. The enzyme-linked immunosorbent assay (ELISA) was used to calculate the Abs concentration, and a calibration curve of Abs was determined by measuring different concentrations of anti-SUs mAbs (0.01-0.065 μg/mL) at the absorbance of 450 nm (Figure S1).

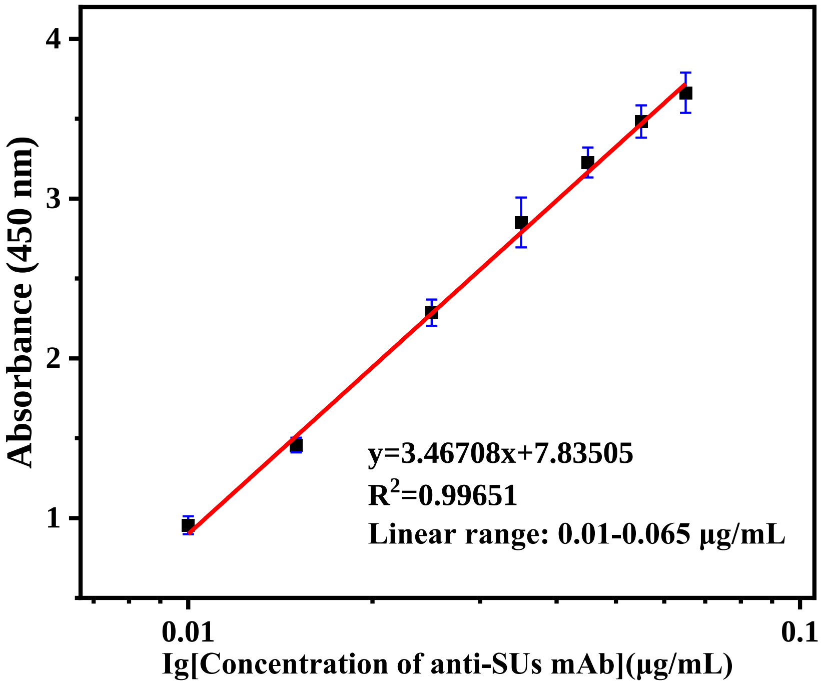

Figure S1. The calibration curve of anti-SUs mAbs.

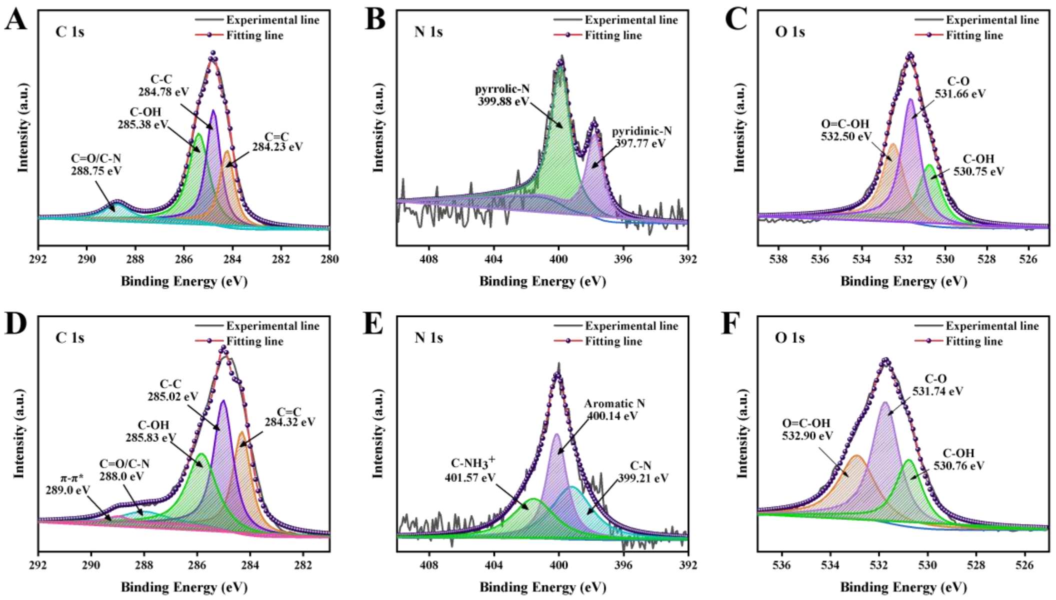

Figure S2. Corresponding XPS high-resolution spectra in the regions of C 1s, N 1s and O 1s. A-C: PCN-224; D-F: PCN-224@PDA. Compared with the PCN-224, the PCN-224@PDA displayed higher or lower binding energy in the regions of C 1s, N 1s, and O 1s. Further, the XPS spectrum in the region of C 1s for PCN-224@PDA showed a new π-π band at 289.0 eV, which was attributed to the aromatic ring of PDA loaded onto the PCN-224 surface through π-π stacking.

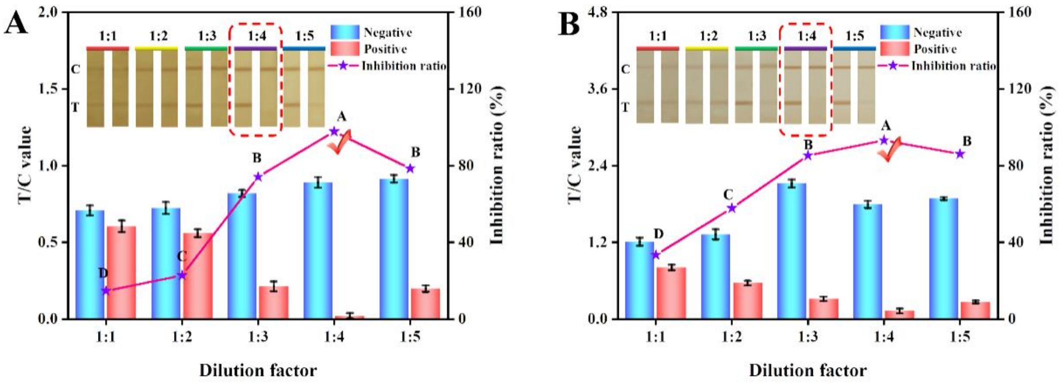

Figure S3. The influence of sample dilution factor on test strip results. A: Tea sample; B: Capsule sample. The concentration of GP was fixed at 45 μg/kg and 70 μg/kg in tea and capsule samples, respectively. Different letters represent statistical differences ($p < 0.01$).

Table S1. Working conditions of the PCN-224@PDA-LFIA

Working conditions	PCN-224@PDA-LFIA
Particle diameter and color of PCN-224	100 nm, dark purple
The polymerization time of PDA	12 h
The amount of DA	12 mg
the pH value for self-polymerization of DA	0.01 M Tris-HCl (pH 7.5)
Particle diameter and color of PCN-224@PDA	120 nm, brown
Concentration of immobilized antigen (SUs-OVA)	0.3 mg/mL
Concentration of goat anti-mouse IgG	0.15 mg/mL
Coating buffer	0.02 M PB (pH 6.5)
The amount of Abs	5 μg
The coupling pH value	0.02 M MES (pH 6.5)
The coupling time of Abs	1 h
Sample pad material	SB08
Sample pad pretreatment solution	0.02 M PB (pH 7.4, 0.75 % Tween®-20, 0.5 % BSA, and 0.3% PVP)
PCN-224@PDA-Abs probes resuspension	0.02 M PB (pH 7.4, 0.5% BSA, 0.5% Tween®-20, 5% sucrose, 0.2% PVP, and 0.03% ProClin^{TM} 300)
Sample pretreatment	1 g of sample was solubilized in 1 mL methanol and shaken for 3 min. After centrifugation at 10000×g for 5 min, the supernatant was filtered through a 0.22 μm organic filter membrane and then diluted five times with 0.02 M PB (pH 7.4).

Table S2. The optimal MS/MS parameters for the detection of SUs

Analyte	Precursor ion (m/z)	Product ion (m/z)	Declustering Potential (eV)	Collision energy (V)
GP	446.2	320.9*	80.00	18.00
	446.2	437.0	80.00	19.13
GM	491.2	352.1*	110.00	17.00
	491.2	125.9	110.00	34.28
GB	494.1	368.9*	90.00	17.00
	494.1	168.8	90.00	38.42
TB	270.9	171.9*	66.00	16.00
	270.9	155.0	66.00	25.89
GQ	528.1	402.9*	93.00	19.00
	528.1	386.1	93.00	28.25
CB	272.3	156.0*	59.00	19.80
	272.3	74.1	59.00	15.00
GL	324.1	110.0*	81.00	24.00
	324.1	127.1	81.00	23.00
AH	325.1	243.0*	94.00	15.00
	325.1	119.0	94.00	34.00
TB	311.9	115*	86.00	22.00
	311.9	91.1	86.00	40.00
CPM	277.0	111.0*	56.40	42.00
	277.0	174.8	50.74	20.00
GBN	367.0	170.2*	63.08	20.00
	367.0	152.1	75.98	26.00

* Quantitative ion

Table S3. Cross-reactivity of icELISA and PCN-224@PDA-LFIA (n = 3)

Analytes	icELISA IC_{50} (μg/kg)	CR%	PCN-224@PDA-LFIA IC_{50} (μg/kg)	CR%
GP	0.15	100.0	5.78	100.0
GM	0.18	83.3	9.05	63.9
GB	0.15	100.0	14.49	39.9
TB	0.65	23.1	31.51	18.3
GQ	0.22	68.2	46.48	12.4
GL	1.17	12.8	141.20	4.1
CB	0.88	17.0	88.99	6.5
TLZ	2.53	5.9	132.54	4.4
AH	3.25	4.6	212.40	2.7
CPM	61.16	0.2	4034.35	0.1
GBN	49.01	0.3	2537.67	0.2
RGLN	＞10,000	＜0.01	＞10,000	＜0.01
RGLT	＞10,000	＜0.01	＞10,000	＜0.01
PF	＞10,000	＜0.01	＞10,000	＜0.01
MFM	＞10,000	＜0.01	＞10,000	＜0.01

Table S4. Determination of SUs in commercial functional tea and capsule samples by PCN-224@PDA-LFIA and LC-MS/MS (n = 3)

Sample No.	LC-MS/MS (μg/kg)	CV%	PCN-224@PDA-LFIA (μg/kg)	CV%
Tea1	NA	-	NA	-
Tea2	NA	-	NA	-
Tea3	NA	-	NA	-
Tea4	NA	-	NA	-
Tea5	NA	-	NA	-
Tea6	NA	-	NA	-
Tea7	NA	-	NA	-
Tea8	NA	-	NA	-
Tea9	NA	-	NA	-
Tea10	NA	-	NA	-
Capsule1	NA	-	NA	-
Capsule2	NA	-	NA	-
Capsule3	NA	-	NA	-
Capsule4	NA	-	NA	-
Capsule5	NA	-	NA	-
Capsule6	NA	-	NA	-
Capsule7	NA	-	NA	-
Capsule8	NA	-	NA	-
Capsule9	NA	-	NA	-
Capsule10	NA	-	NA	-
